# Methylmercury Poisoning Induces Cardiac Electrical Remodeling and Increases Arrhythmia Susceptibility and Mortality

**DOI:** 10.3390/ijms21103490

**Published:** 2020-05-15

**Authors:** Mara Cristina P. Santos Ruybal, Monica Gallego, Thais Bazoti B. Sottani, Emiliano H. Medei, Oscar Casis, Jose Hamilton M. Nascimento

**Affiliations:** 1 Instituto de Biofísica Carlos Chagas Filho, Universidade Federal do Rio de Janeiro, 21941-902 Rio de Janeiro, Brazil; mararuybal.fisio@gmail.com (M.C.P.S.R.); thais.bazoti@hotmail.com (T.B.B.S.); emedei70@hotmail.com (E.H.M.); jhmnasc55@hotmail.com (J.H.M.N.); 2 Departamento de Fisiología, Facultad de Farmacia, Universidad del País Vasco UPV/EHU, 01006 Vitoria, Spain; monica.gallego@ehu.eus

**Keywords:** mercury, cardiac, electrical remodeling, ion current, arrhythmia

## Abstract

This study aims to investigate the cardiac electrical remodeling associated with intoxication by methylmercury (MeHg). We evaluated the chronic effects of MeHg on in vivo electrocardiograms and on ex vivo action potentials and depolarizing (I_Ca-L_) and repolarizing (I_to_) currents. The acute effect of MeHg was evaluated on HEK293 cells expressing human ERG, Kv4.3 and KCNQ1/KCNE1 channels. Chronic MeHg treatment increased QTc and T_peak_–T_end_ interval duration, prolonged action potential duration and decreased amplitude of I_to_ and I_Ca-L_. In addition, heterologously expressed I_hKv4.3_, I_hERG_ or I_hKCNQ1/KCNE1_ decreased after acute exposure to MeHg at subnanomolar range. The introduction of the in vitro effects of MeHg in a computer model of human ventricular action potentials triggered early afterdepolarizations and arrhythmia. In conclusion, cardiac electrical remodeling induced by MeHg poisoning is related to the reduction of I_to_ and I_Ca-L_. The acute effect of MeHg on hKv4.3; hERG and hKCNQ1/KCNE1 currents and their transposition to in silico models show an association between MeHg intoxication and acquired Long QT Syndrome in humans. MeHg can exert its high toxicity either after chronic or acute exposure to concentrations as low as picomolar.

## 1. Introduction

Some metals found in nature are essential for life, whereas others have no biologic significance beyond their toxic potential. Heavy metals such as mercury (Hg), cadmium (Cd), arsenic (As) and lead (Pb) are the main chemical contaminants in the environment and can cause organic dysfunctions such as neurological, renal, immunological or teratogenic disorders [1,2,3].

Mercury can cause deleterious effects on human health, which depend on the chemical form, amount, route of exposure and differences in vulnerability among exposed subjects [4,5,6,7]. The main target organs for Hg toxicity are brain [8], myocardium [9], liver and kidney [10], skin [11], lung [12], testis and prostate [13], which are associated with dysfunction after either acute or chronic exposure.

The organic form of mercury, the methylmercury (MeHg), is the most toxic chemical form of Hg and it is widely distributed in the environment, which can affect human health mainly through consumption of contaminated food [14,15]. At the cardiac level, MeHg contamination associates with changes in heart rate variability [16], which is described as a situation that generates risk of cardiac arrhythmias and sudden death [17,18,19,20,21]. In addition, abnormalities in the ST segment and in the T wave of the electrocardiogram (ECG) followed by death from cardiac arrest caused by organic Hg poisoning were described decades ago [22]. However, as far as we know, further studies on the ECG of Hg exposed people have not been subsequently made.

Even though several evidences strongly suggest the potential cardiac toxicity of MeHg, the basis and the mechanism underlying are poorly understood yet. Therefore, the aim of this study was to investigate whether acute or chronic methylmercury poisoning may promote cardiac electrical remodeling with arrhythmogenic potential. We evaluated electrocardiographic parameters in vivo, action potentials in cardiac preparations ex vivo and ionic currents in rat cardiomyocytes and HEK293 cells in vitro. Finally, transposition of the results to in silico models shows an association between MeHg intoxication and prolonged action potential and arrhythmias in humans. Taken together the results obtained here support the hypothesis that MeHg impairs the cardiac electrical functions and increase the arrhythmic probability.

## 2. Results

### 2.1. MeHg Treatment Induced Weight Loss and Higher Mortality

After 4 weeks of treatment, no significant changes were observed in the control group, whereas the MeHg-treated group showed substantial weight loss (312 ± 6 vs. 261.4 ± 13.3 g in control vs. MeHg, respectively, *p* < 0.01) and mortality (0/21 vs. 11/30 death animals in control vs. MeHg, respectively, *p* < 0.05).

Although the absolute weight of heart and liver was reduced after 4 weeks of MeHg treatment, the relative weight was changed in neither heart, liver nor lungs when normalized to the total body weight or tibia length.

Taken together these results depict that MeHg treatment increases mortality and impairs the energetic metabolism since drastically decreased the normal weight gain of the treated rats.

### 2.2. QTc and APD90 Were Longer in the MeHg-Treated Group

**Figure 1:**
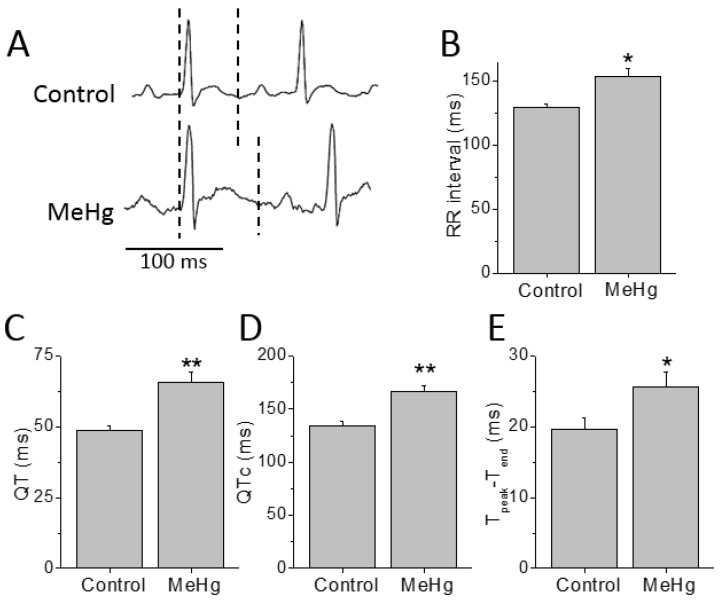
Chronic MeHg treatment prolongs cardiac repolarization in vivo. (**A**) Electrocardiograms of a conscious animal before and 4 weeks after treatment with MeHg 3 mg/kg·day (dashed lines show the QT interval). RR interval (**B**), QT interval, (**C**) heart rate corrected QT interval, QTc, (**D**) and repolarization dispersion, T_peak_–T_end_ (**E**) were prolonged after MeHg treatment. *n* = 13 control and 15 MeHg-treated animals. * *p* < 0.05; ** *p* < 0.01.

Electrocardiographic recordings were performed before and after 4 weeks of treatment with MeHg (Figure 1). Compared to control, MeHg-treated rats had prolonged QT interval after 4 weeks treatment. The ECGs of MeHg-treated rats also showed a prolongation of the RR interval compared to control animals. In order to overcome the effects of the frequency on QT interval (the time between the beginning of the QRS complex and the endo of the T wave) duration it was corrected to the heart rate. Thus, the corrected QT interval (QTc) values were also higher in MeHg-treated animals when compared to the age-matched control group. The T_peak_–T_end_ interval is another electrocardiographic risk marker of arrhythmic and mortality outcomes and it was also significantly prolonged in MeHg-treated animals (Figure 1).

**Figure 2:**
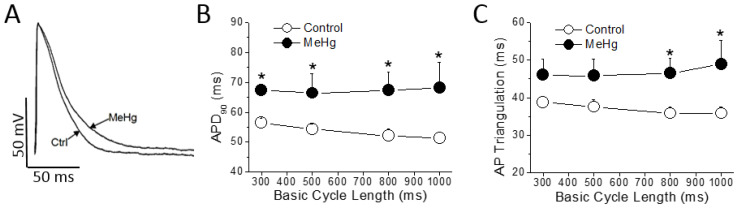
MeHg prolongs ventricular action potential duration. (**A**) ventricular action potentials were recorded in epicardial strips from control and MeHg-treated animals; (**B**) action potential duration at 90% of repolarization (APD_90_) and (**C**) action potential (AP) triangulation at different basic cycle length (BCL), in control (open symbols) or after 4 weeks of treatment with MeHg (filled symbols). * *p* < 0.05. *n* = 7 control and 14 MeHg-treated hearts per group.

The ECG changes seen in MeHg-treated animals represent a substrate for the development of cardiac arrhythmias, so the cellular mechanisms of these alterations must be explored. In order to identify the mechanisms responsible for the alterations found in the electrocardiogram of MeHg-treated animals, the action potentials of ventricular muscle strips were recorded (Figure 2 and Supplementary Table S1).

MeHg treatment had no effect either on resting membrane potential, on AP amplitude or on maximum upstroke velocity (dV/dt). However, also as observed in the ECG, MeHg prolonged the ventricular repolarization at the cellular level significantly increasing the APD_90_. This prolongation was evident at any Basic Cycle Length (BCL) tested (Figure 2B). The AP triangulation, other important parameter accepted as a pro-arrhythmogenic marker, was also prolonged in a statistically significant manner at higher BCLs of stimulation (Figure 2C).

Collectively, these data support the idea that MeHg induces strong electrical changes consistently associated with cardiac pro-arrhythmogenic events: lengthened the QTc, increased the T_peak_–T_end_ and prolonged the APD_90_.

### 2.3. MeHg Prolongs the Cardiac Ventricular Repolarization by Decreasing I_to_ Current

**Figure 3:**
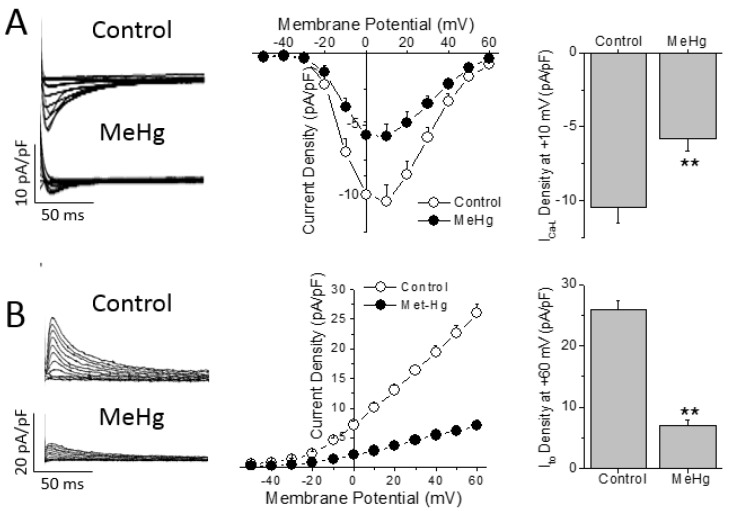
MeHg treatment reduces the transient outward K^+^ and the L-type Ca^2+^ currents in ventricular myocytes. (**A**) I_Ca-L_ currents were recorded at voltages from −50 to +60 mV in ventricular myocytes isolated from animals after 4 weeks in control conditions or treated with MeHg. Current–voltage relationship obtained in myocytes from animals treated with vehicle (open symbols, *n* = 7) or with MeHg (filled symbols, *n* = 6). (**B**) I_to_ traces recorded at potentials between −50 and +60 mV in ventricular myocytes isolated from animals after 4 weeks in control conditions or treated with MeHg. Current–voltage relationship obtained in myocytes from animals treated with vehicle (open symbols, *n* = 18) or with MeHg (filled symbols, *n* = 14). ** *p* < 0.01.

The AP shape and duration is determined by a fine balance between the outward repolarizing currents (i.e., transient outward potassium current—I_to_) and the inward depolarizing currents (i.e., L-type Ca^2+^ current, I_Ca-L_). Thus, the differences in AP duration can be due to changes in either outward or inward currents. Chronic treatment with MeHg induced a ~44% reduction of the I_Ca-L_ density in comparison with control non treated animals (Figure 3A). However, no changes were found in the biophysical properties measured here, as observed by the similar values obtained in both activation and inactivation half voltages (V_h_) in both groups studied (Supplementary Table S2).

While I_Ca-L_ is the main depolarizing current that determines the action potential duration in rats, I_to_ is the pivotal repolarizing current that regulates the action potential duration in rats. Four weeks of treatment with MeHg reduced the amplitude of I_to_ in a statistically significant manner in comparison with control animals at all voltages over +10 mV (Figure 3B). When we explored the biophysical properties of the I_to_, it was observed that MeHg shifted both the activation and inactivation half voltages to more negative potentials. Finally, recovery from I_to_ inactivation was slowed by MeHg treatment, as can be seen in the larger recovery time constant (Supplementary Table S2).

### 2.4. Methylmercury Decrease the Key Human Repolarizing Cardiac Currents

All results demonstrated that MeHg induced cardiac electrical remodeling and the main molecular mechanisms in vivo in rats. Next, a set of experiments were performed in order to assess whether MeHg could change the density and/or the characteristics of the main repolarizing currents in the human heart: the I_to_, carried by Kv4.3 channels; I_Kr_, carried by hERG or Kv11.1 channels; and I_Ks_, carried by the combination of Kv7.1 channels and the accessory protein MinK.

**Figure 4:**
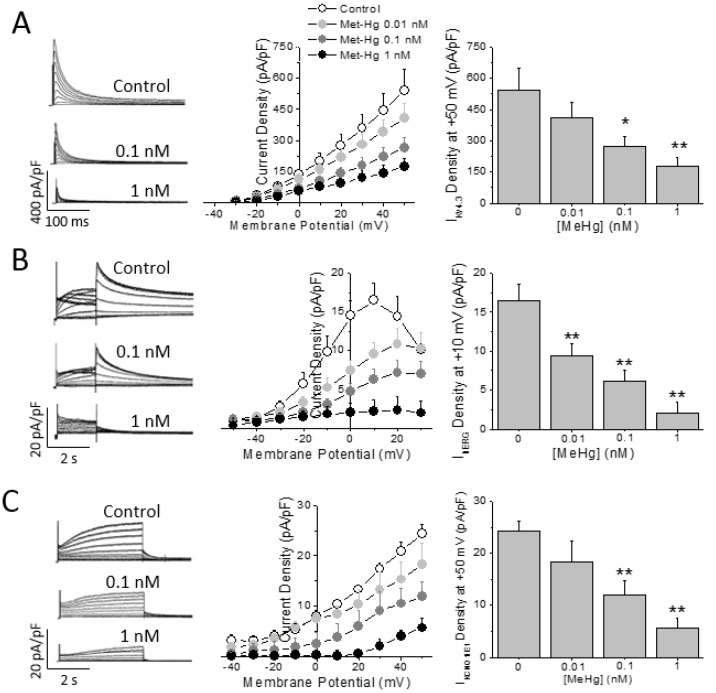
MeHg reduces repolarization capability in human cells in vitro. Representative traces and average current density-voltage relationships obtained in HEK293 cells expressing (**A**) Kv4.3 channels, (**B**) hERG channels, or (**C**) Kv7.1/KCNE1 channels. MeHg acute exposure reduces all currents in a concentration dependent manner. *n* = 8–15 cells. * *p* < 0.05; ** *p* < 0.01.

HEK293 cells expressing the human cardiac K^+^ channels alpha subunit Kv4.3 were challenged to MeHg at different concentrations. As observed in Figure 4A MeHg was able to reduce in a concentration dependent manner (from 0.01 to 1 nM) the I_Kv4.3_ density. On the other hand, neither the inactivation half voltage nor the recovery from inactivation time constant were changed by the acute exposure to MeHg (Supplementary Table S3).

The main repolarizing current in the human ventricle is I_Kr_, carried by the Kv11.1 or hERG channel protein. Thus, the I_hERG_ in HEK293 cells stably expressing hERG channels was recorded in the presence of different MeHg concentrations. Acute exposure to MeHg reduced I_hERG_ current amplitude in a concentration dependent manner (Figure 4B). The current was reduced by a concentration as low as 0.01 nM and almost disappeared at concentration of 1 nM. Neither the activation half voltage nor the reversal potential of I_hERG_ were changed by any concentration of MeHg (Supplementary Table S3).

Finally, HEK293 cells were transfected with both the Kv7.1 channel alpha subunit and the accessory protein KCNE1 or MinK, which assemble to form the I_Ks_ conducting channel. The I_Ks_ was recorded and exposed to increasing concentrations of MeHg. I_Ks_ was reduced in a concentration dependent manner (Figure 4C). In addition, the current properties were modulated by MeHg perfusion at different concentrations. Thus, MeHg shifted the activation half voltage to more positive potentials (Supplementary Table S3).

### 2.5. MeHg Is Pro-Arrhythmic in Human Ventricle in Silico

The data obtained in human ion currents clearly suggest that MeHg is able to negatively modulate the key ventricular repolarizing potassium currents. Thus, the potential arrhythmogenic effect of MeHg was tested in silico.

**Figure 5:**
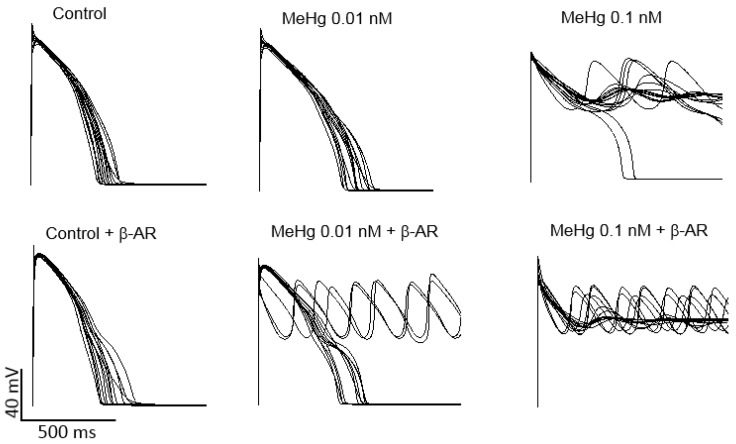
Modeling the effects of acute MeHg-induced changes in ion currents on human ventricular action potential. Computational models were generated to simulate action potential of 100 control individuals in resting conditions and under β-adrenergic stimulation. For clarity, only the first 15 traces are shown. When the ionic conductances were changed to simulate the effects of 0.01 nM MeHg AP duration increases in resting conditions, but after β-adrenergic stimulation, 33% of action potentials generated early afterdepolarizations and arrhythmia. When the ionic conductances were changed to simulate the effects of 0.1 nM MeHg 98% models showed early afterdepolarizations (EADs) and arrhythmia in resting conditions and 100% after β-adrenergic stimulation.

Ventricular AP models of 100 different healthy humans with different ion channel densities were generated either in resting conditions or after β-adrenergic stimulation. Then, the results on the percentage of the current reduction obtained in human HEK293 cells transfected with K^+^ channels present in the human ventricle were introduced in the models in order to establish a similar simulated condition. When ionic conductance of each channel was changed to simulate an exposure to 0.01 nM MeHg, it can be seen a prolongation of APD, but no arrhythmia was observed. It is widely accepted that when a reduction of key potassium ion channels prolongs APD, a β-adrenergic stimulation could act as a trigger of ventricular arrhythmias. In the present work, adding a β-adrenergic stimulation after MeHg exposure early after depolarization (EADs) and arrhythmias appeared in 30% of the models (Figure 5). As can be also seen in Figure 5, MeHg at a concentration of 0.1 nM induced EADs and arrhythmia in almost all simulated individuals in resting conditions.

## 3. Discussion

Thousands of workers from mercury mines and mills were studied along the 20th century in Europe [23]. The study concluded that the mortality was higher among these workers when compared with people with different works in the same countries. The higher mortality in people chronically exposed to Hg was associated with cardiovascular diseases such as hypertension, cardiac ischemia or cardiac stroke [23,24]. In addition to the chronic effects, acute Hg exposure has been directly related to sudden death, in some cases from unknown origin and others from cardiac arrest [22,25].

In the present work, chronic MeHg exposure reduced the cardiac rhythm, prolonged the rate corrected ventricular repolarization time (QTc) and increased ventricular repolarization dispersion (T_peak_–T_end_), parameters associated with increased risk of cardiac arrhythmia. Despite of the high mortality observed in human populations exposed to Hg, we found no studies regarding the effects of this heavy metal on human cardiac electrophysiology. There are, however, electrocardiographic studies with arsenic, another heavy metal, in leukemic patients treated with As_2_O_3_ and in general population chronically exposed to arsenic contaminated water [26,27]. Interestingly, these works report similar electrocardiographic abnormalities than those we observe in rats chronically exposed to MeHg.

Since the electrocardiogram is the result of the summation of the action potentials of every cardiac cell, we explored the effects of in vivo chronic MeHg exposure on the AP characteristics on ventricular strips from treated and untreated rats. As expected, in cardiac strips from hearts isolated from animals with prolonged QTc, the ventricular repolarization was also prolonged at the cellular level. The APD_90_ and the AP triangulation were longer when compared to control animals. The increase in cardiac AP duration could be due to an increase of the depolarizing Ca^2+^ current I_Ca-L_, a decrease of the repolarizing K^+^ currents or both. In the present work, both I_Ca-L_, and I_to_ were reduced after chronic in vivo exposure to MeHg. This reduction in the two currents at the same time can explain the absence of effect on APD_30_, since this parameter reflects the duration of the plateau phase of the AP. During this phase, these two currents balance one to each other and the reduction in both of them at the same time maintains the equilibrium. Since I_Ca-L_ is predominant in the initial phase of the plateau, a decrease in the plateau duration could be expected. However, the great reduction in I_to_ is not able to rapidly repolarize the cell even in the condition of reduced I_Ca-L_. Overall, as reflected in the APD_30_, at the initial phase of the plateau there is neither a prolongation nor a shortening. At the end of the plateau, I_Ca-L_ is inactivated, and I_to_ is the predominating current, resulting in a prolongation of the APD_90_. There are no studies in the literature about the effects of in vivo MeHg exposure on cardiac Ca^2+^ or K^+^ currents, but similar reductions were observed in calcium and potassium currents of rat dorsal root ganglion neurons [28,29].

Next, we wanted to explore whether the effect observed in rat I_to_ could also be reflected on the main I_to_ alpha subunit in human, Kv4.3. Acute in vitro exposure to MeHg of cells expressing the hKv4.3 channels induced a concentration dependent reduction of the I_hKv4.3_ with no effect on the biophysical properties of the current. In contrast, chronic in vivo treatment with MeHg reduced the I_to_ current, but also shifted both activation and inactivation half voltages and slowed the current recovery from inactivation. These differences between in vivo and in vitro effects could be due to an effect of MeHg on the channel regulation. One limitation of the study is that in cardiac myocytes I_to_ carries through Kv4.3 channels associated with auxiliary subunits such as KChIP2, whereas in HEK293 cells the current was carried out only through Kv4.3 channels. MeHg in vivo could affect the association of the channel with accessory proteins or their intracellular signaling pathway regulation. Thus, MeHg decreases I_to_ current and thus prolongs repolarization providing a cellular substrate for arrhythmia. Additionally, the results obtained in the human Kv4.3 alpha subunit strongly suggest that MeHg exerts a direct modulation of the channel.

The main repolarizing current in the human ventricle is the rapid delayed rectifier potassium current I_Kr_. This current is carried through the Kv11.1 or hERG channel. The data showed a concentration dependent reduction of the I_hERG_ in the subnanomolar range. This result is similar to that found after exposure of these channels to other heavy metals such as cobalt or arsenic [30,31].

The slow delayed rectifier K^+^ current, I_Ks_, is responsible for the adaptation of the AP duration to different heart rates and adrenergic stimulation. This current is carried through a channel formed by the Kv7.1 alpha subunit with the regulatory protein KCNE1. In vitro exposure induced a concentration dependent reduction of I_Ks_ and a positive shift in the voltage dependence of activation. These results also are in agreement with those obtained after Kv7.1/KCNE1 exposure to arsenic [31].

Finally, in order to translate the experimental data obtained on human K^+^ channels regarding the susceptibility to trigger arrhythmia to human hearts, we used computer simulations of human cardiac action potentials. When the effect of 0.01 nM MeHg on each current was introduced in the models, the action potential duration increased in resting conditions, in accordance with the prolongation of the QT interval and APD_90_ observed in treated rats and consistent with the reduction of the amplitude of the repolarizing K^+^ currents. It is important to note that in the AP models, EADs and arrhythmias emerged only under β-adrenergic stimulation, also consistent with the reduction observed in I_Ks_.

In conclusion, MeHg induces ventricular electrical remodeling prone to cardiac arrhythmias. MeHg can exert its high toxicity either after chronic or acute exposure to concentrations as low as the picomolar range.

## 4. Materials and Methods

### 4.1. Animals and Experimental Protocol

All animal experiments comply with the National Institutes of Health guide for the care and use of Laboratory animals (NIH Publications No. 8023, revised 1978). All experimental procedures were approved by the Committee for Ethics in Animal Experimentation of the Federal University of Rio de Janeiro (protocol N° IBCCF-109 and 029/14). Male Wistar rats (200–250 g) were used in this study. The animals were kept in boxes maintained at an approximate temperature of 23 °C, 12 h light/dark cycle and access to food and water ad libitum. The animals were randomly distributed in two experimental groups, named MeHg and Ctrl group, respectively, which received daily treatment, for 28 days by gavage, with a solution containing 3 mg/kg·day of methylmercury chloride (CH_3_HgCl) or equivalent volume of vehicle (MilliQ water). The dose and duration of treatment was selected because it is in the medium range of dosage described to induce symptoms of MeHg intoxication such as decreased mobility and ataxia [32,33,34].

At the end of experimental period, the animals were subjected to biometric and electrocardiographic evaluation in vivo. Then, the animals were heparinized (500 IU/kg of sodium heparin, Sigma-Aldrich, Sant Louis, MO, USA) intraperitoneal and euthanized by cervical dislocation under anesthesia with isoflurane.

### 4.2. Biometric Analysis

After euthanasia, the lungs, liver and heart were removed and weighed using the same balance in which the body weight was measured (Marte A500, São Paulo, SP, Brazil). The ratios between heart, liver and lung weights with either body weight or tibia length were calculated to establish the indirect rates of cardiac hypertrophy, venous congestion and pulmonary congestion, respectively.

### 4.3. In Vivo Electrocardiogram

Electrocardiogram recording was carried out in conscious animals by a non-invasive method. Electrodes were positioned in DII derivation and connected by flexible cables to a differential AC amplifier (Model 1700, A-M Systems, USA) with signal low-pass filtered at 500 Hz. The signal was digitized at 1 kHz sample rate by a 16-bit A/D converter (Minidigi 1-B, Molecular Devices, San Jose, CA, USA) using the Axoscope 10 software (Molecular Devices, San Jose, CA, USA). Data were stored in a PC for offline processing.

### 4.4. Action Potential (AP) Recording

Left ventricle preparations were used to assess the action potential profile. Muscle strips (≈0.5 cm) were cut and pinned to the bottom of a tissue bath. The preparations were perfused with Tyrode’s Solution containing (in mM): NaCl 140; KCl 5; MgCl_2_ 1.8; 11 glucose; HEPES 5; CaCl_2_ 2 (pH 7.4 adjusted with NaOH at 37 °C) saturated with oxygen (100%) at a constant flow of 5 mL/min with a peristaltic pump (Miniplus 3, Gilson, USA). The tissue was stimulated at four different basic cycle lengths (BCLs) 1000, 800, 500 and 300 ms using field stimulation. Transmembrane potential was recorded using glass microelectrodes filled with 3 M KCl, connected to a high input impedance microelectrode amplifier (Electro 705, WPI, USA). Amplified signals were digitized (1440 Digidata A/D interface, Molecular Devices, San Jose, CA, USA) and stored in a computer for later analysis using the software LabChart 7 (AD Instruments, Bella Vista, New South Wales, Australia). Afterwards, the resting membrane potential (RMP), action potential amplitude (APA), maximum speed of depolarization (dV/dt) and action potential duration at 90% (APD_90_) and 30% (APD_30_) of the repolarization were analyzed. The action potential triangulation was calculated by subtracting APD_30_ from APD_90_.

### 4.5. Cardiomyocytes Isolation

Following the treatment period, the animals were heparinized and euthanized. The hearts were quickly excised by thoracotomy, weighed and cannulated by the aorta in a modified Langendorff apparatus for retrograde perfusion with oxygenated Tyrode’s solution (in mM): NaCl 140; KCl 4; MgCl_2_ 1; HEPES 10; glucose 10; and CaCl_2_ 2; pH adjusted to 7.4 with NaOH at 37 °C in a constant 10 mL/min flow rate. After 5 min, perfusion solution was changed to nominally Ca^2+^-free Tyrode’s solution. After the hearts stopped beating, type II collagenase (Worthington Biochemical, NJ, USA) was added to Ca^2+^-free Tyrode’s solution in a 0.5 mg/mL concentration and recirculated for 8 min. To complete digestion, the hearts were washed with Ca^2+^-free Tyrode’s solution for 20 min. The epicardium of the left ventricles was dissected, minced and gently shaken to dissociate cardiomyocytes. Isolated cells were stored in modified KB solution (in mM): KCl 30; glutamic acid 70; KH_2_PO_4_ 10; MgCl_2_ 1; taurine 20; HEPES 10; glucose 10; EGTA 0.3; pH = 7.3 with KOH at room temperature (23–25 °C).

### 4.6. HEK293 Cell Culture and Transfection

Human Embryonic Kidney cells (HEK293) and HEK293 cell lines stably expressing hERG channel (HEK-hERG) or Kv.4.3 channel (HEK-Kv4.3) were cultured in DMEM (Dubelcco’s modified Eagle’s medium), supplemented with 10% of fetal bovine serum and 1% of penicillin-streptomycin-amphotericin cocktail. HEK-hERG or HEK-Kv4.3 grew in the presence of 50 μM of geneticin. Cell cultures were maintained in 5% CO_2_ at 37 °C. HEK293 cells were transfected with plasmids containing genes related to human ion channel I_Ks_. The transfections into HEK293 cells of 1 µg/mL, 0.5 µg/mL and 0.2 µg/mL of plasmid containing cDNA of KCNQ1, KCNE1 (2:1 ratio) and GFP, respectively, was performed using Lipofectamine 2000 (Invitrogen) according to the instruction of the manufacturer.

### 4.7. Patch–Clamp Recordings in Cardiomyocytes

Isolated myocytes were transferred to a shallow chamber and allowed to settle for at least 30 min before being superfused with the external bathing solution (Tyrode’s solution with nicardipine 10 µM for I_to_ or without nicardipine for I_Ca-L_). Only Ca^2+^-tolerant rod-shaped cells, with clear cross-striations and lacking any visible blebs on their surfaces were used. All experiments were performed at room temperature (23–25 °C). Ionic currents were recorded using the whole-cell configuration of the patch–clamp technique with an Axopatch-1D patch–clamp amplifier (Axon Instruments, Inc., USA). Recording pipettes were obtained from borosilicate tubes (Sutter Instruments, Novato, CA, USA) and had a tip resistance of 2–5 MΩ when filled with the internal solution (in mmol/L): CsCl 110; TEACl 30; Mg-ATP 5; EGTA 10; HEPES 10 and Na-GTP 0.1 to record the L-type Ca^2+^ current, I_Ca-L_ or KCl 125; NaCl 10; MgCl_2_ 5; HEPES 10 and EGTA 5 to record the transient outward K^+^ current, I_to_.

Following the patch rupture, whole cell membrane capacitances were measured from integration of the capacitive transients elicited by voltage steps from −70 mV to −75, −65 and −60 mV, which did not activate any voltage-dependent membrane current. For both I_to_ and I_Ca,L_ recording, voltage pulses ranging from −50 to +60 mV, starting from a holding potential of −60 mV preceded by pre-pulse to −40 mV were applied and current amplitudes were normalized to cell capacitance and expressed as pA/pF. Steady-state activation curve of I_to_ or I_ca-L_ was obtained from the I–V ratio, calculating the conductance (G) to the K^+^ or Ca^2+^ ion, respectively.

To evaluate the voltage dependence of the I_to_ stationary inactivation (Steady-state inactivation), a double-pulse protocol was used ranging from −90 to +30 mV, starting from a holding potential of −80 mV followed by pulse to +60 mV. Recovery from inactivation of I_to_ was assessed by a double-pulse protocol from −80 to +60 mV, where the time between two consecutive pulses was variable.

To evaluate the voltage dependence of the I_Ca-L_ steady-state inactivation, a double-pulse protocol was used ranging from −90 to +40 mV, starting from a holding potential of −80 followed by a pulse to 0 mV.

### 4.8. Patch–Clamp Recordings in HEK293 Cells

HEK293 cells plates were superfused with the external bathing solution containing (in mmol): NaCl 136; KCl 4; CaCl_2_ 1.8; MgCl_2_; HEPES-Na 10 and glucose 10 (pH 7.4) with or without methylmercury chloride (CH_3_HgCl) that was used in 0.01 nM, 0.1 nM and 1 nM concentrations. All experiments were performed at room temperature (21–22 °C). Ionic currents were recorded using the whole-cell configuration of the patch–clamp technique with an Axopatch-200B patch–clamp amplifier (Axon Instruments, Inc., USA). Recording pipettes were obtained from borosilicate tubes (Sutter Instruments, USA) and had a tip resistance of 1–3 MΩ when filled with the internal solution (in mmol/L): KCl 125; MgCl_2_ 5; EGTA-K 5; HEPES-K 10; ATP-Na 5 (pH 7.2). Following the patch rupture, whole cell membrane capacitances were measured from integration of the capacitive transients elicited by voltage steps from −50 to −60 mV, which did not activate any voltage-dependent membrane current. Currents amplitudes were normalized to cell capacitance and expressed as pA/pF.

For HEK-hERG recording, voltage pulses ranging from −80 to +50 mV starting from a holding potential of −90 mV followed by a post-pulse to −40 mV, were applied. Steady-state activation curve of HEK-hERG was obtained from the I–V ratio of tail current. To evaluate the fully activated HEK-hERG current, a double-pulse protocol was used ranging from −120 to +60 mV, preceded by a pulse to +40 mV starting from a holding potential of −90 mV.

For KCNQ1/KCNE1 recording in transfected HEK293 cells, voltage pulses ranging from −40 to +50 mV starting from a holding potential of −80 mV were applied. Steady-state activation curve of KCNQ1/KCNE1 was obtained from the I–V ratio.

For HEK-Kv4.3 recording, voltage pulses ranging from −30 to +50 mV starting from a holding potential of −80 mV were applied. Steady-state inactivation curve of HEK-K_V_4.3 a double-pulse protocol was used ranging from −100 to +10 mV, starting from a holding potential of −80 mV followed by a pulse to +50 mV. Inactivation recovery of Kv4.3 channels was assessed by a double-pulse protocol, where the time between the two pulses from −80 to +50 mV was variable.

### 4.9. Computational Modeling

Human ventricular action potentials of 100 control individuals were simulated using the O’Hara–Rudy dynamic (ORd) model as baseline [35]. Ionic conductances were varied between 20–200% of the O’Hara–Rudy “average human” to generate models with different ion current profiles keeping the AP parameters within the normal range [36]. The simulations presented were conducted using Virtual Assay, Copyright© 2016, University of Oxford (v.1.3.640 2014 Oxford University Innovation, Ltd., Oxford, UK), a software package for in silico drug assays, kindly provided by B. Rodríguez.

In order to reproduce the effects of MeHg in this population of 100 AP models, ionic conductances were modified according to our experimental findings in human ion channels in human cells. Trains of 300 APs were evoked at 1 Hz and the last AP trace in each simulation was presented. The simulations were carried out either in resting conditions or after β-adrenergic stimulation.

### 4.10. Statistical Analysis

Data are presented as mean ± SEM. Comparisons between groups were performed using Student’s *t*-test or one-way analysis of variance, followed by a Bonferroni multiple comparison test. Values of *p* < 0.05 were considered statistically significant. All analyses were performed using GraphPad Prism 5.0 (GraphPad Software, USA).

## Supplementary Materials

Supplementary materials can be found at https://www.mdpi.com/1422-0067/21/10/3490/s1.

## References

[1] Wolf M.B., Baynes J.W. (2007). Cadmium and mercury cause an oxidative stress-induced endotelial dysfunction. BioMetals.

[2] Zukowska J., Biziuk M. (2008). Methodological evaluation of method for dietary heavy metal intake. J. Food Sci..

[3] Singh R., Gautam N., Mishra A. (2011). Heavy metals and living systems: An overview. Indian J. Pharmacol..

[4] Clarkson T.W. (1972). The pharmacology of mercury compounds. Annu. Rev. Pharmacol..

[5] Möller-Madsen B. (1994). Localization of mercury in CNS of the rat. Pharmacol. Toxicol..

[6] Guzzi G., La Porta C.A. (2008). Molecular mechanisms triggered by mercury. Toxicology.

[7] Kim K.H., Kabir E., Jahan S.A. (2016). A review on the distribution of Hg in the environment and its human health impacts. J. Hazard Mater..

[8] Yoshida M., Honda A., Watanabe C., Satoh M., Yasutake A. (2014). Neurobehavioral changes in response to alterations in gene expression profiles in the brains of mice exposed to low and high levels of mercury vapor during postnatal development. J. Toxicol. Sci..

[9] Vassallo D.V., Simões M.R., Furieri L.B., Fioresi M., Fiorim J., Almeida E.A., Angeli J.K., Wiggers G.A., Peçanha F.M., Salaices M. (2011). Toxic effects of mercury, lead and gadolinium on vascular reactivity. Braz. J. Med. Biol. Res..

[10] Joshi D., Mittal D.K., Shukla S., Srivastav A.K., Srivastav S.K. (2014). N-acetyl cysteine and selenium protects mercuric chloride-induced oxidative stress and antioxidant defense system in liver and kidney of rats: A histopathological approach. J. Trace Elem. Med. Biol..

[11] Moody R.P., Joncas J., Richardson M., Petrovic S., Chu I. (2009). Contaminated soils (II): In vitro dermal absorption of nickel (NI-63) and mercury (Hg-203) in human skin. J. Toxicol. Environ. Health. A.

[12] Lim H.E., Shim J.J., Lee S.Y., Lee S.H., Kang S.Y., Jo J.Y., In K.H., Kim H.G., Yoo S.H., Kang K.H. (1998). Mercury inhalation poisoning and acute lung injury. Korean J. Intern. Med..

[13] Martinez C.S., Torres J.G., Peçanha F.M., Anselmo-Franci J.A., Vassallo D.V., Salaices M., Alonso M.J., Wiggers G.A. (2014). 60-Day chronic exposure to low concentrations of HgCl2 impairs sperm quality: Hormonal imbalance and oxidative stress as potential routes for reproductive dysfunction in rats. PLoS ONE.

[14] Chen C.Y., Driscoll C.T., Lambert K.F., Mason R.P., Sunderland E.M. (2016). Connecting mercury science to policy: From sources to seafood. Rev. Environ. Health.

[15] Li P., Du B., Chan H.M., Feng X., Li B. (2018). Mercury bioaccumulation and its toxic effects in rats fed with methylmercury polluted rice. Sci. Tot. Environ..

[16] Gribble M.O., Cheng A., Berger R.D., Rosman L., Guallar E. (2015). Mercury Exposure and Heart Rate Variability: A Systematic Review. Curr. Environ. Health Rep..

[17] Talman N.T. (1985). Cardiovascular regulation lesions of the central nervous system. Ann. Neurol..

[18] Oppenheimer S.M., Cechetto D.F., Hachinski V.C. (1990). Cerebrogenic cardiac arrhythmias. Arch. Neurol..

[19] Mayer S.A., Fink M.E., Homma S., Sherman D., LiMandri G., Lennihan L., Solomon R.A., Klebanoff L.M., Beckford A., Raps E.C. (1994). Cardiac injury associated with neurogenic pulmonary edema following subarachnoid hemorrhage. Neurology.

[20] Marmar V., Kalyanam S. (2008). The role of the autonomic nervous system in sudden cardiac death. Prog. Cardiovasc. Dis..

[21] Huang C.L.H. (2017). Murine Electrophysiological Models of Cardiac Arrhythmogenesis. Physiol. Rev..

[22] Cinca I., Dumitrescu I., Onaca P., Serbanescu A., Nestorescu B. (1979). Accidental ethyl mercury poisoning with nervous system, skeletal muscle, and myocardium injury. J. Neurol. Neurosurg. Psychiatry.

[23] Boffetta P., Sallsten G., Garcia-Gomez M., Pompe-Kirn V., Zaridze D., Bulbulvan M., Caballero J.D., Ceccarelli F., Kobal A.B., Merler E. (2001). Mortality from cardiovascular diseases and exposure to inorganic mercury. Occup. Environ. Med..

[24] Salonen J.T., Seppanen K., Nyyssonen K., Korpela H., Kauhanen J., Kantola M., Tuomilehto J., Esterbauer H., Tatzber F., Salonen R. (1995). Intake of mercury from fish, lipid peroxidation, and the risk of myocardial infarction and coronary, cardiovascular, and any death in eastern Finnish men. Circulation.

[25] Eisler R. (2003). Health risks of goldminers: A synoptic review. Environm. Geochem. Health.

[26] Roboz G.J., Ritchie E.K., Carlin R.F., Samuel M., Gale L., Provenzano-Gober J.L., Curcio T.J., Feldman E.J., Klingfield P.D. (2014). Prevalence, management, and clinical consequences of QT interval prolongation during treatment with arsenic trioxide. J. Clin. Oncol..

[27] Chen Y., Wu F., Parvez F., Ahmed A., Eunus M., Mcclintock T.R., Patwary T.I., Islam T., Ghosal A.K., Islam S. (2013). Arsenic exposure from drinking water and QT Interval prolongation: Results from the Health Effects of Arsenic Longitudinal Study. Environ. Health Perspect..

[28] Leonhardt R., Haas H., Busselberg D. (1996). Methylmercury reduces voltage-activated currents of rat dorsal root ganglion neurons. Naunyn Schmiedebergs Arch. Pharmacol..

[29] Leonhardt R., Pekel M., Platt B., Haas H., Busselberg D. (1996). Voltage-activated calcium channel currents of rat DRG neurons are reduced by mercuric chloride (HgCl2) and methylmercury (CH3HgCl). Neurotoxicology.

[30] Sanchez-Chapula J.A., Sanguinetti M.C. (2000). Altered gating of HERG potassium channels by cobalt and lanthanum. Pflugers. Archiv..

[31] Drolet B., Simard C., Roden D.M. (2004). Unusual effects of a QT-prolonging drug, arsenic trioxide, on cardiac potassium currents. Circulation.

[32] Fujimura M., Usuki F., Kawamura M., Izumo S. (2011). Inhibition of the Rho/ROCK pathway prevents neuronal degeneration in vitro and in vivo following methylmercury exposure. Toxicol. Appl. Pharmacol..

[33] Li Y., Fan Y., Zhao J., Xu X., Jing H., Shang L., Gao Y., Li B., Li Y.F. (2016). Elevated mercury bound to serum proteins in methylmercury poisoned rats after selenium treatment. Biometals.

[34] Liu Y., Ji J., Zhang W., Suo Y., Zhao J., Lin X., Cui L., Li B., Hu H., Chen C. (2019). Selenium modulated gut flora and promoted decomposition of methylmercury in methylmercury-poisoned rats. Ecotoxicol. Environ. Saf..

[35] O’Hara T., Virág L., Varró A., Rudy Y. (2011). Simulation of the undiseased human cardiac ventricular action potential: Model formulation and experimental validation. PLoS Comput. Biol..

[36] Passini E., Britton O.J., Lu H.R., Rohrbacher J., Hermans A.N., Gallacher D.J., Greig R.J.H., Bueno-Orovio A., Rodriguez B. (2017). Human in Silico Drug Trials Demonstrate Higher Accuracy than Animal Models in Predicting Clinical Pro-Arrhythmic Cardiotoxicity. Front. Physiol..

