# Supplementary material for: Methylmercury Poisoning Induces Cardiac Electrical Remodeling and Increases Arrhythmia Susceptibility and Mortality
Source: Int J Mol Sci. 2020 May 15;21(10):3490. doi: 10.3390/ijms21103490 (PMC7279040; doi:10.3390/ijms21103490)
Supplement: Supplementary file 1 [file ijms-21-03490-s001.pdf]

Table 1. Action Potential Characteristics in hearts from control and MeHg treated animals for 4 weeks. n = 8-14 animals.*$p<0.05$. RP, resting membrane potential; APA, action potential amplitude; APD_{30}, action potential duration at 30% of repolarization; APD_{90}, action potential duration at 90% of repolarization; Triang, action potential triangulation (APD_{90}-APD_{30}); dV/dt, maximum upstroke velocity.

	**Basic Cycle Length Duration (ms)**							
	**300**		**500**		**800**		**1000**	
	**Cntrl (14)**	**MeHg (7)**	**Cntrl (13)**	**MeHg (8)**	**Cntrl (12)**	**MeHg (9)**	**Cntrl (12)**	**MeHg (8)**
**RP (mV)**	-75.7 ± 0.9	-75 ± 0.9	-78 ± 2	-76 ± 1.2	-75 ± 0.7	-75 ± 1	-74 ± 0.7	-76 ± 1
**APA (mV)**	90.4 ± 3.4	87 ± 2.4	89.3 ± 2.6	88.8 ± 1.8	91.3 ± 2.5	89 ± 1.4	89.8 ± 1.9	90.3 ± 1.3
**APD_{30} (ms)**	17.6 ± 1.1	21.2 ± 2	16.9 ± 1	20.6 ± 2.5	16.3 ± 0.9	20.9 ± 2.6	15.5 ± 0.8	19.3 ± 2.4
**APD_{90} (ms)**	56.5 ± 1.4	67.3 ± 5.2*	54.4 ± 1.7	66.4 ± 6.6*	52.1 ± 2.3	67.3 ± 6*	51.3 ± 2	68.2 ± 8.5*
**Triang (ms)**	38.8 ± 1.6	46 ± 4.1	37.5 ± 1.9	45.8 ± 4.5	35.8 ± 1.8	46.5 ± 4.1*	35.8 ± 1.8	48.9 ± 6.4*
**dV/dt (V/ms)**	109.2 ± 11	110 ± 22	126 ± 19	155 ± 42	111 ± 10	141 ± 46	109 ± 10	144 ± 47

Table 2. Biophysical characteristics of I_{Ca-L} and I_{to} in control and in MeHg treated animals for 4 weeks. Number of cells in parenthesis. *p < 0.05.

	**Control**	**MeHg**
$\mathbf{I_{Ca-L}}$ Activation V_h (mV)	-7.05 ± 1.5 (7)	-8.5 ± 2.3 (6)
$\mathbf{I_{Ca-L}}$ Inactivation V_h (mV)	31.5 ± 5 (7)	30.1 ± 1.9 (6)
$\mathbf{I_{to}}$ Activation V_h (mV)	5.1 ± 2.9 (18)	1.8 ± 2.4* (14)
$\mathbf{I_{to}}$ Inactivation V_h (mV)	-44.7 ± 1.7 (12)	-49.2 ± 0.8* (13)
$\mathbf{I_{to}}$ $\tau_{recovery}$ (ms)	63.6 ± 9.1 (7)	174.2 ± 44.4* (7)

Table 3. Biophysical characteristics of hKv4.3, hERG and hKv7.1/mink channels in HEK293 cells in control and in the presence of increasing concentrations of MeHg. Number of cells in parenthesis. *p < 0.05. (///) hKv7.1/mink tail currents were not detectable in the presence of 1 nM of MeHg.

	**[MeHg] (nM)**			
	**0**	**0.01**	**0.1**	**1**
$I_{hKv4.3}$ Inactivation V_h (mV)	-56.8 ± 1.6 (12)	-54.9 ± 1.5 (7)	-58.5 ± 1.3 (6)	-58.5 ± 1.3 (5)
$I_{hKv4.3}$ Recovery τ (ms)	150.7 ± 12.9 (6)	191.5 ± 25 (6)	169 ± 13.9 (8)	190 ± 22.4 (6)
I_{hERG} ActivationV_h (mV)	-3.1 ± 2.1 (8)	3.9 ± 1.1 (9)	0.5 ± 1.4 (12)	-6.7 ± 1.8 (5)
I_{hERG} Reversal V (mV)	-66.5 ± 2.3 (10)	-60.6 ± 2.2 (14)	-61.1 ± 1.1 (14)	-68.5 ± 3.3 (5)
$I_{hK7.1/minK}$Activation V_h (mV)	10.2 ± 2 (6)	16.9 ± 2.2 (6)	26.9 ± 6.1 (7)*	///
